# Biomarker discovery by integrated joint non-negative matrix factorization and pathway signature analyses

**DOI:** 10.1038/s41598-018-28066-w

**Published:** 2018-06-27

**Authors:** Naoya Fujita, Shinji Mizuarai, Katsuhiko Murakami, Kenta Nakai

**Affiliations:** 1 Human Genome Center, the Institute of Medical Science, the University of Tokyo, Tokyo, Japan; 2 Discovery and Preclinical Research Division, Taiho Pharmaceutical Co., Ltd., Tsukuba, Japan; 3 Department of Computational Biology and Medical Sciences, Graduate school of Frontier Sciences, the University of Tokyo, Kashiwa, Japan

## Abstract

Predictive biomarkers are important for selecting appropriate patients for particular treatments. Comprehensive genomic, transcriptomic, and pharmacological data provide clues for understanding relationships between biomarkers and drugs. However, it is still difficult to mine biologically meaningful biomarkers from multi-omics data. Here, we developed an approach for mining multi-omics cell line data by integrating joint non-negative matrix factorization (JNMF) and pathway signature analyses to identify candidate biomarkers. The JNMF detected known associations between biomarkers and drugs such as *BRAF* mutation with PLX4720 and *HER2* amplification with lapatinib. Furthermore, we observed that tumours with both *BRAF* mutation and MITF activation were more sensitive to BRAF inhibitors compared to tumours with *BRAF* mutation without MITF activation. Therefore, activation of the BRAF/MITF axis seems to be a more appropriate biomarker for predicting the efficacy of a BRAF inhibitor than the conventional biomarker of *BRAF* mutation alone. Our biomarker discovery scheme represents an integration of JNMF multi-omics clustering and multi-layer interpretation based on pathway gene signature analyses. This approach is also expected to be useful for establishing drug development strategies, identifying pharmacodynamic biomarkers, in mode of action analysis, as well as for mining drug response data in a clinical setting.

## Introduction

Precision medicine for cancer patients with molecular targeted drugs and predictive biomarkers is expected to lead to a paradigm shift from one-size-fits-all medicine to patient-specific medicine^1^. In particular, in the era of cancer immunology, the immunohistochemistry of programmed death ligand-1 (PD-L1) expression levels has been approved as a companion diagnostic for the anti-PD-1 antibody pembrolizumab; however, the identification of useful biomarkers remains a significant issue^2^. Selecting appropriate patients for a particular treatment using predictive biomarkers will certainly help to increase therapeutic effectiveness and reduce toxicities. Thus, it is important to identify reliable predictive biomarkers to select the right patient for the right drug.

Comprehensive genomic and pharmacological data of large collections of cancer cell lines have been published as the Cancer Cell Line Encyclopedia (CCLE)^3,4^. These cell line databases provide mutation, copy number alteration, and mRNA expression profiles, as well as the results of tests of the sensitivities of cells to growth inhibition induced by various compounds or drugs. Although there is some discordance between databases, especially in terms of the compound sensitivity profiles, these databases generally show reasonable consistency^5,6^.

These multi-dimensional genomic and pharmacological datasets have been used to perform multi-omics analyses with the goal of understanding the relationships between cancer genomes and drug responders. The NCI DREAM challenge is an example of an approach leading to remarkable improvements in this area^7^, in which several prediction models were proposed to estimate sensitivity to compounds based on genetic information. The top-performing method was found to be a kernel method with multiview and multitask learning, which uses all of the genetic profiles provided^7^. Although this challenge is focused on providing a benchmarked set of algorithms, it is difficult to translate the results obtained from the predictors for clinical application. This is because the models simultaneously require genetic, epigenomic, and proteomic data, and such comprehensive models make for challenging biological interpretations. Moreover, other research programs have focused on discovering useful biomarker candidates in clinical settings rather than relying on predictive performance. Several well-known biomarker and drug associations were detected using analysis of variance (ANOVA)- or regression-based analyses from multi-omics data^3,4^. However, some biological features such as tissue-specific expression are correlated, which poses a limitation in the reliability of ANOVA with multiplicity and regression analysis with multicollinearity in handling these features. Furthermore, the most common type of predictive biomarker measured clinically with a companion diagnosis kit is a single gene that is equivalent to the therapeutic target itself or a gene that is biologically relevant to the target^8^. Therefore, it is still challenging to efficiently utilize comprehensive genomic data to determine an appropriate treatment strategy. In this study, we sought to resolve these issues to facilitate the use of multi-omics analyses for understanding relationships between the cancer genome and drug responders through development of comprehensive prediction models with multi-genetic features. Since the choice of predictive biomarkers for suggesting treatment options to patients should be based on a biological rationale, we focused on detecting biologically meaningful biomarkers rather than merely developing comprehensive multi-omics predictors.

Non-negative matrix factorization (NMF) is an unsupervised approach that can highlight outliers or extreme characteristics in a non-negative input matrix *X* according to its parts-based representation nature^9^. Matrix *X* is then factorized by the non-negative submatrices *W* and *H*. NMF has emerged as one of the most useful algorithms currently available in the cancer genome research field. An NMF method was used to generate mutational signatures for 96 trinucleotide mutation patterns from the genomes of cancer patients^10,11^. For example, this method detected that smoking-related C > A mutations at NpCpN and POLE mutations were related to C > A and T > G mutations in a TpCpT and TpTpT context, respectively. Thus, a mutational signature or NMF approach can help to decompose the multiple effects of a carcinogen based on a patient’s combined mutation pattern. Furthermore, NMF can classify tumour subtypes from microarray data. Indeed, NMF was used to identify a small number of gene combinations (metagenes) whose profiles represent features that can distinguish among leukaemia and lung cancer subtypes^12^. NMF is essentially applicable for a single-input matrix such as face imaging pixels and mutational signature profiles, although it has potential to be further extended to multiple inputs. Joint non-negative matrix factorization (JNMF) is fitted for multiple inputs with the same row size, which generates a common sub matrix *W* and individual sub matrices *H*s^13^. JNMF can therefore be used to detect common clusters (co-modules) from mRNA expression, microRNA expression, and DNA methylation data of cancer patients. Thus, we hypothesized that JNMF would be a suitable method to handle several multi-omics datasets simultaneously. Moreover, among the many techniques available to handle multiple inputs^14^, JNMF is theoretically and practically equivalent to a standard NMF method with concatenated inputs.

With respect to resolving the biological interpretation challenge, pathway analysis can be a useful tool to annotate a given set of genes in a biologically meaningful manner^15^. Ingenuity Pathway Analysis (IPA) is one of the most beneficial tools available to understand the association of various types of molecules, and provides causal networks based on biological relationships curated from the literature^16^. Furthermore, gene signature analysis enables inferences on pathway activation and dependency^17,18^. Therefore, pathway and gene signature analyses are effective for understanding particular gene sets identified as NMF co-modules.

**Figure 1:**
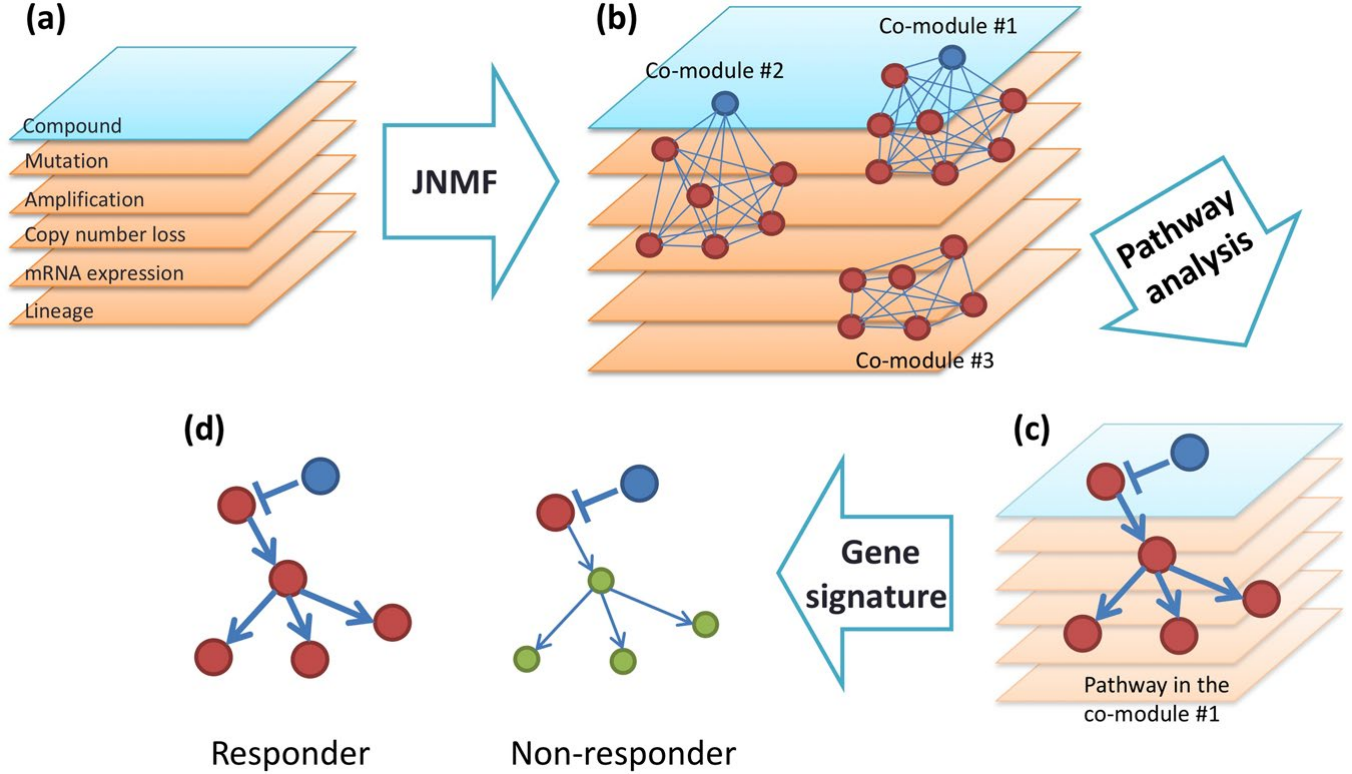
Biomarker discovery scheme. (**a**) Input matrices are multi-dimensional pharmacological, genomic, transcriptomic, and tumour-type data aligned by cell lines. (**b**) The joint non-negative matrix factorization (JNMF) method detects multi-dimensional co-modules. Each module shows the co-occurrence between genetic (red) and pharmacological (blue) features. (**c**) Pathway analysis provides causal relationship based on biological knowledge in co-module features. (**d**) Gene signature analysis clarifies the relationship between pathway activation and sensitivity to compounds.

Our research objective was to identify promising candidate biomarkers using multi-dimensional genomic and pharmacological data from a collection of cell lines. The approach used is based on the integration of JNMF multi-omics clustering with multi-layer interpretation based on pathway gene signature analyses (Fig. 1). This scheme enabled us to identify novel rationale-based biomarkers as well as known clinically validated biomarkers.

## Results

### JNMF with missing data

To illustrate the robustness of our multi-omics clustering method against missing values, JNMF was first applied to simulated data. Three simulated input matrices were generated: a simulated compound sensitivity matrix, simulated mutation matrix, and simulated mRNA expression matrix. Four co-modules were predefined in these simulated matrices, in line with a previous report^13^. To best mimic real data, the simulated genetic mutation matrix was represented in a binary format, whereas the simulated compound sensitivity and mRNA expression matrices were represented in a continuous format. The simulated compound sensitivity matrix contained certain missing values randomly (10%), because typical sensitivity metrics (IC_**50**_, GI_50_, AUC, etc.) often fail due to the lack of measurement of a compound in a cell line or after filtering out noisy results. The simulated matrices of mutation and expression profiles contained missing rows randomly (10%), since public genomic and transcriptomic data cannot always be fully assigned to all cell lines for compound sensitivity data.

**Figure 2:**
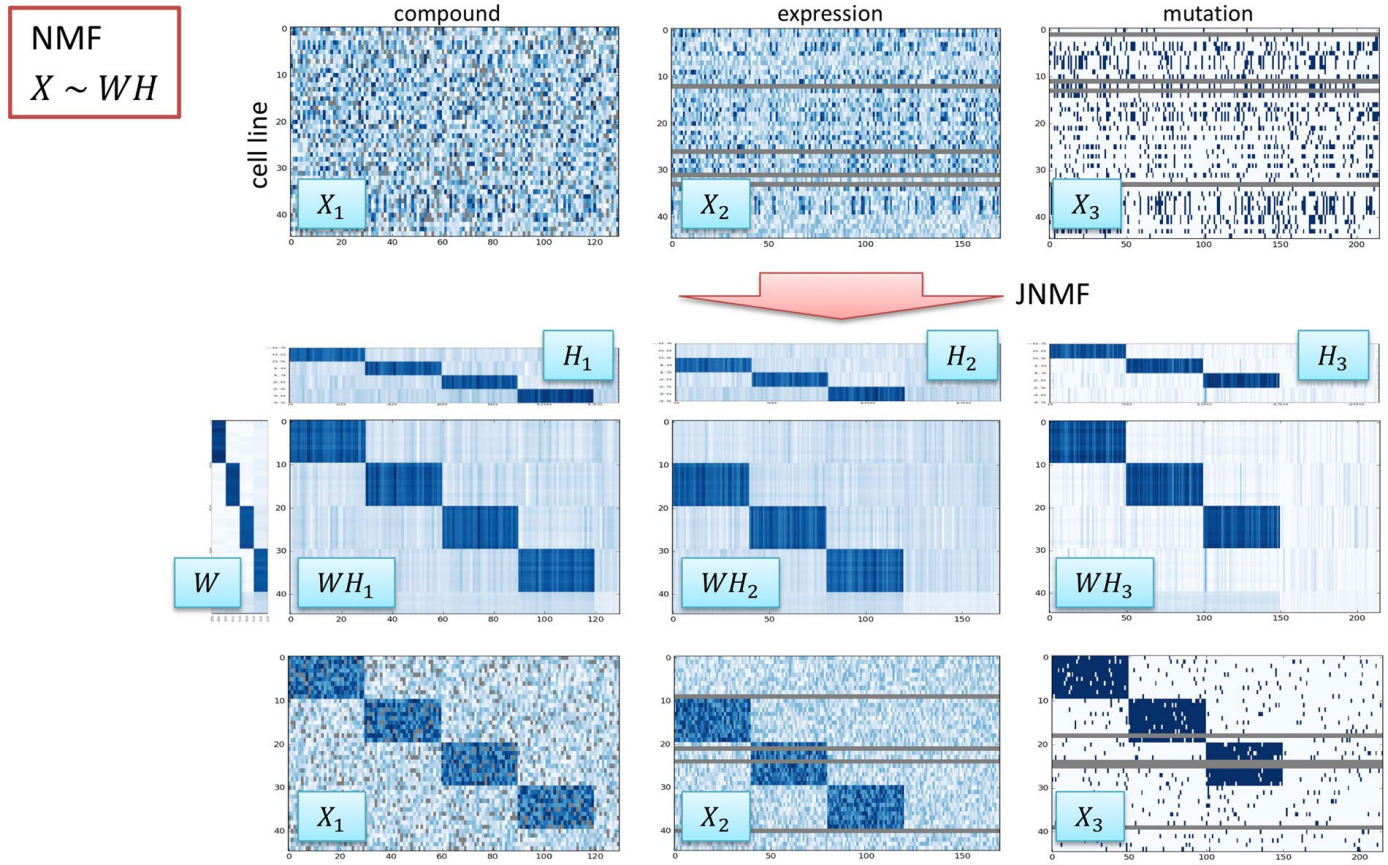
JNMF multi-dimensional clustering with simulated data. Simulated compound, expression, and mutation data are clustered using JNMF to detect co-modules. The simulated compound dataset in continuous format ***X***_1_ contains four modules as well as missing components. The simulated expression dataset in continuous format ***X***_2_ contains three modules as well as missing profiles in rows. The simulated mutation dataset in binary format ***X***_3_ contains three modules as well as missing profiles in rows. Continuous ***X***_1_ and ***X***_2_ have Gaussian noise, and binary ***X***_3_ is partially flip-flopped as noise. JNMF generates meta-profiles ***H***_1_, ***H***_1_, ***H***_3_, and **W**.

JNMF for the three simulated matrices with factorization rank k = 4 correctly revealed the four predefined clusters (Fig. 2). JNMF with *X*_1_, *X*_2_, and *X*_3_ inputs returned *H*_1_, *H*_2_, *H*_3_, and *W* submatrices. *WH*_1_ clearly reproduced *X*_1_, resulting in four modules (blue submatrices), and *WH*_2_ and *WH*_3_ contained three modules. Therefore, JNMF detected co-clusters hidden in the input matrices despite the presence of missing values. The dimensionality reduction effect of JNMF also results in a noise reduction effect. Furthermore, JNMF could interpolate the missing values. This property of JNMF might enable predicting the mutation status from expression profiles, as well as predicting compound sensitivity from genomic profiles. However, further experimental validation must be conducted to validate these applications.

We next investigated the effects of missing and noise values on the results of JNMF. For the same simulated datasets, the following four parameters were systematically examined by a 0.1 grid to check the consistency of elements in the four co-modules: *m* as the missing rate of all input *X*s, and *a*, *b*, and *c* as the noise rates of *X*_1_, *X*_2_, and *X*_3_, respectively. Regression analysis revealed that the consistency of the co-module in *W*, *y*_*W*_, is affected by the parameters *m*, *a*, and *c* (Fig. S1a), whereas the other consistency indexes *y*_*H1*_, *y*_*H2*_, and *y*_*H3*_ are affected by {*m*, *a*}, {*m*, *b*}, and {*m*, *c*}, respectively (Fig. S1b–d). In this artificial case, a missing ratio *m* = 0.1 had a slight influence on the results.

### Detection of known biomarkers by JNMF

To discover the relationship between the compound sensitivity and multi-omics profiles of cell lines, we conducted JNMF with factorization rank ***k*** = **40** for the CCLE data set^3^ as follows: compound sensitivity ***X***_**1**_, mutation ***X***_**2**_, copy number amplification ***X***_**3**_, copy number loss ***X***_**4**_, mRNA expression ***X***_**5**_, and tumour type ***X***_**6**_ (Table S1). JNMF was applied to find 40 meta-profiles for cell lines as a ***W*** matrix and 40 co-modules for genetic and pharmacological features as ***H*** matrices simultaneously. We repeated 10 trials of JNMF with random initial values. The results showed that the objective function sufficiently converged in all 10 trials (Fig. S2). Furthermore, a consensus matrix for ***W*** showed high concordance between trials with a cophenetic correlation coefficient of 0.91 (Fig. S3). This consensus matrix contained robust clusters with melanoma, blood cancer, and hypermutated profiles. This consistency reflects the fact that similar JNMF clusters could be obtained for any initial condition. Thus, JNMF would be able to detect unique characteristics from multi-dimensional genomic and pharmacologic data. The best result was selected as that showing the smallest objective function value among the 10 trials, which was used for further analysis.

**Figure 3:**
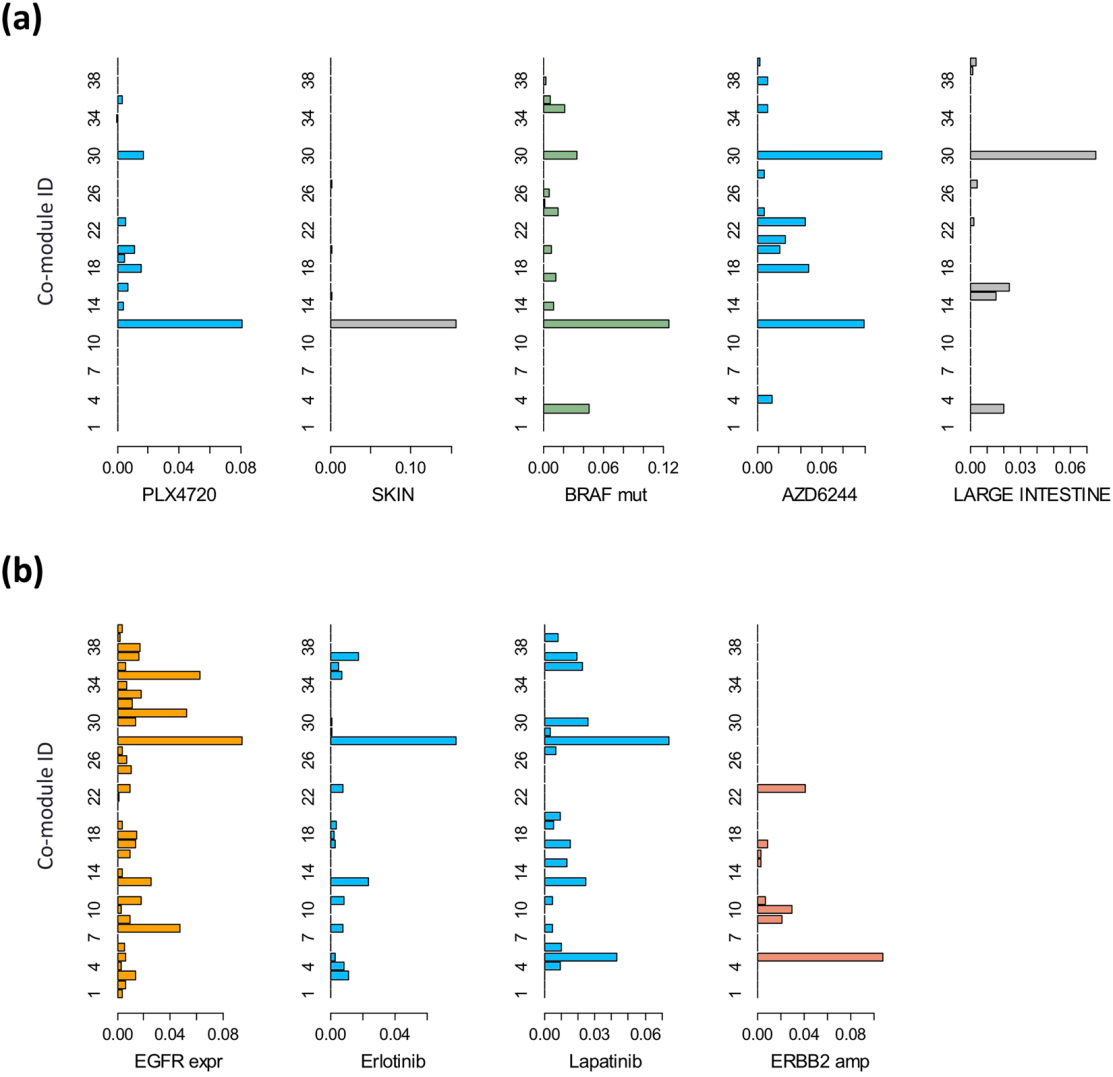
Differences in co-modules between compounds and related biomarkers. (**a**) Difference between sensitivity profiles to PLX4720 and AZD6244. The x-axis shows JNMF meta-profile levels and the y-axis shows 40 JNMF co-module IDs. Co-module #12 is enriched with both PLX4720 and AZD6244, and co-module #30 is an AZD6244-specific co-module. (**b**) Difference between sensitivity profiles to erlotinib and lapatinib. Co-module #28 is enriched with both erlotinib and lapatinib, and co-module #5 is a lapatinib-specific co-module.

JNMF revealed several well-established relationships between drugs and biomarkers. For example, *BRAF*-mutated melanoma cells and patients in preclinical and clinical trials show specific sensitivity to the BRAF inhibitors PLX4720 and its structural analogue, PLX4032 (vemurafenib)^19,20^. In line with this knowledge, our JNMF result returned a co-module (#12) that contained PLX4720, *BRAF* mutation, and melanoma tumour type (Fig. 3a). Another co-module (#5) was related to human epidermal growth factor receptor 2 (HER2)-activated breast tumours, which was enriched with the features of HER2 amplification, overexpression, and breast tumour, and also showed sensitivity to the HER2 inhibitor lapatinib (Fig. 3b). Ultimately, we selected a factorization rank of 40 based on high consistency of the JNMF results with biologically useful knowledge. Furthermore, each genetic or pharmacological feature belonged to one co-module according to the consensus and connectivity matrices scheme. In addition to the HER2-breast cancer (#5) and BRAF–melanoma (#12) co-modules, other cancer-related co-modules were selected, such as those representing the hypermutated phenotype (#3) and responders to receptor tyrosine kinase inhibitors (#28) (Table S2).

### Pathway analysis predicted activation of microphthalmia-associated transcription factor (MITF) in the melanoma cluster

To interpret the biological relationships in the obtained multi-omics JNMF co-module clusters, pathway analysis was performed for the most highly expressed gene sets in the co-modules to investigate whether or not a specific pathway was activated. IPA upstream analysis estimated module-specific upstream regulators, including transcription factors (Table S2), and suggested that the transcription factor MITF was activated in co-module #12, which was highly significant among co-modules (IPA overlap p-value = 6.91E-39, Fig. S4). Therefore, we focused on co-module #12, which was sensitive to BRAF inhibition and included strong melanoma characteristics, and was also one of the highly reproducible modules among the 10 trials as mentioned above. Furthermore, IPA protein-protein interaction (PPI) analysis for co-module #12 showed that melanoma-related transcription factors, such as MITF, PAX3, SOX8, and SOX10, formed a sub-network (Fig. S5). Thus, these analyses of upstream regulator estimation and PPI network elucidated *MITF* activation. Since co-module #12 also included *MITF* amplification and overexpression of *MITF* itself, it was presumed that the gene sets were highly expressed as a result of *MITF* activation associated with *MITF* amplification. Therefore, it seems that *MITF* amplification and activation is a characteristic of this module.

### MITF activation signature as a novel predictive biomarker for BRAF inhibition

**Figure 4:**
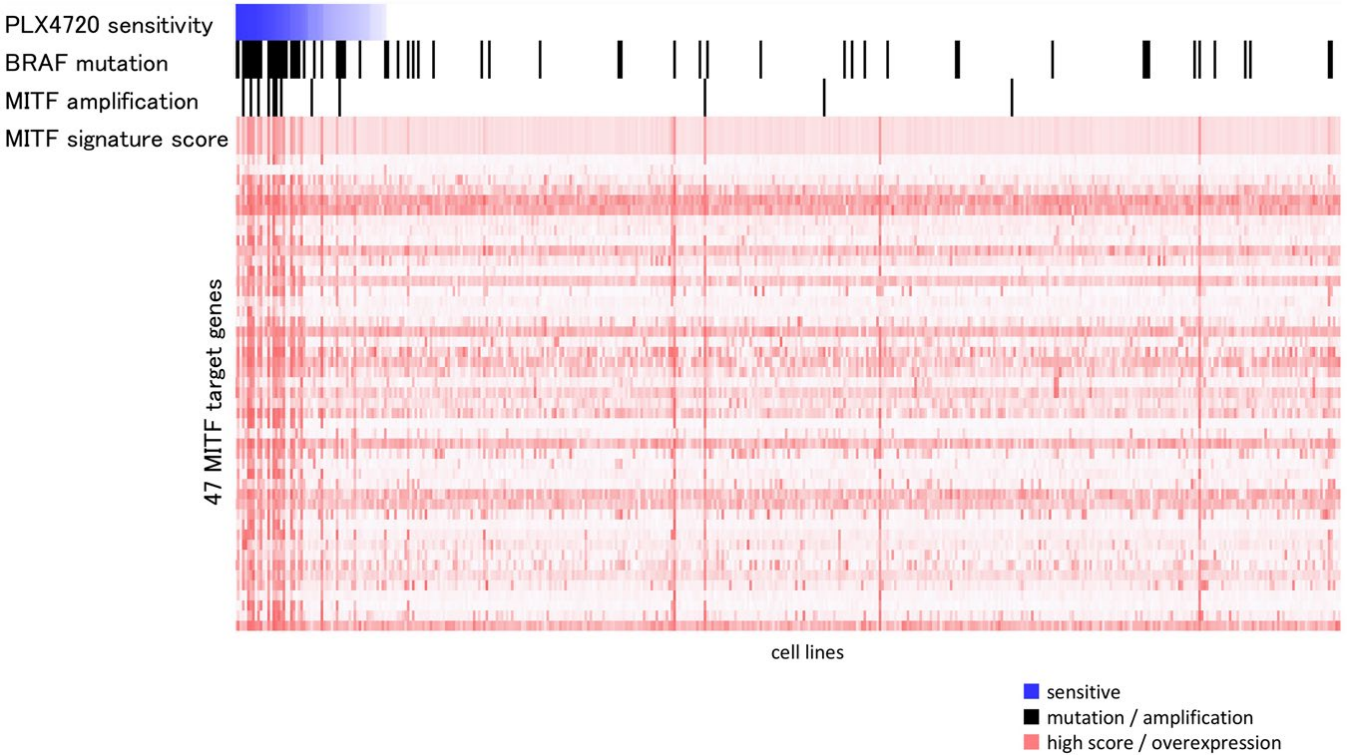
MITF-BRAF in the input data. Correlation between the PLX4720 sensitivity profile and alteration of the BRAF/MITF pathway in the JNMF input set. The x-axis shows cell lines according to their PLX4720 sensitivity. The y-axis represents the PLX4720 sensitivity profiles, *BRAF* mutation, *MITF* amplification, MITF activation signature, and expression profiles of the MITF target genes. The score of the MITF activation signature is defined as the averaged and normalized expression profiles of the MITF target genes. JNMF selected these features as belonging to the *BRAF* mutation and PLX4720 cluster.

**Figure 5:**
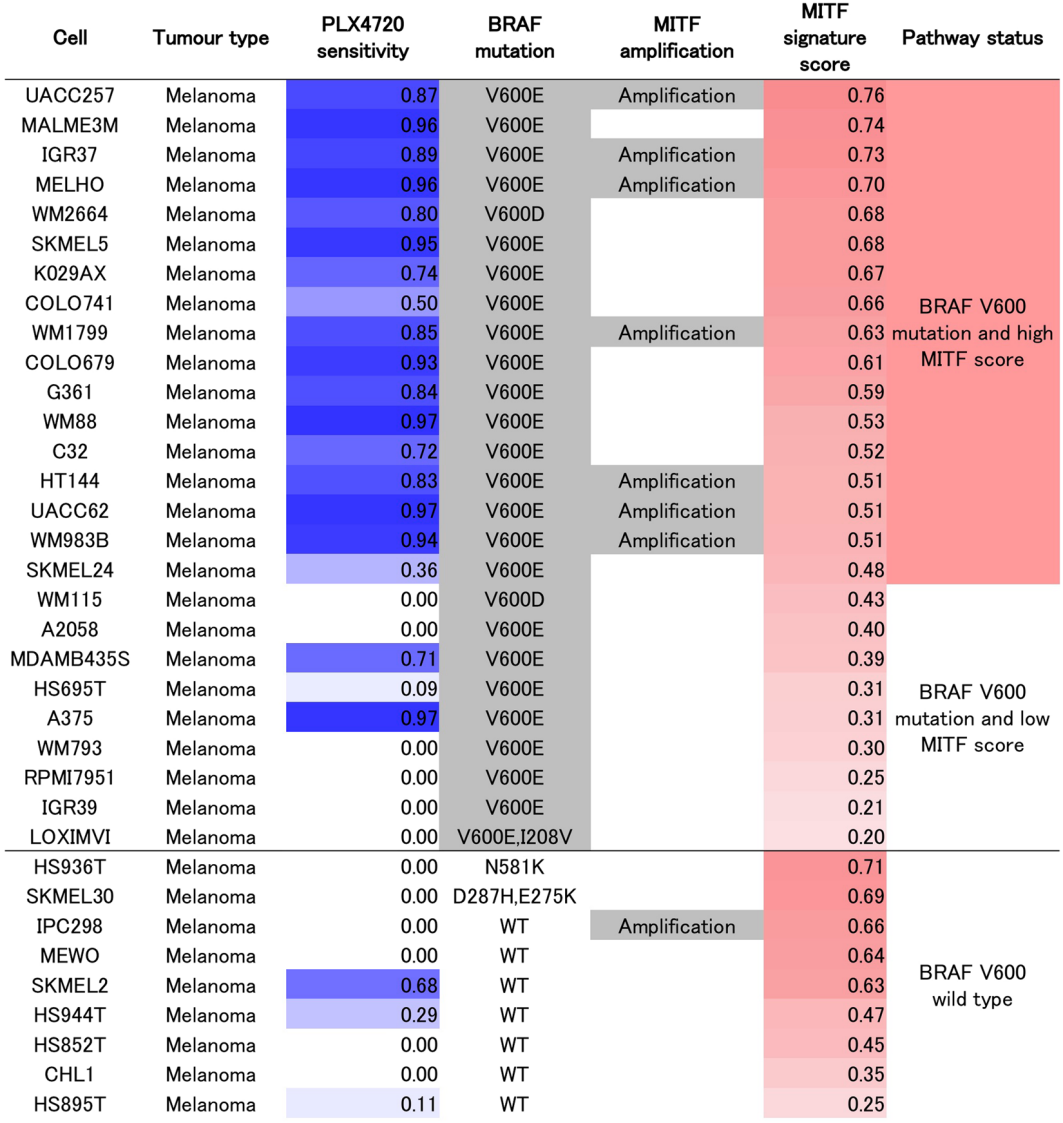
PLX4720 sensitivity profile and alteration of the BRAF/MITF pathway in melanoma cells. The melanoma cells are listed according to their PLX4720 normalized sensitivity profiles (1: sensitive, 0: insensitive), *BRAF* mutation status as reported in the COSMIC database, *MITF* copy number amplification as predicted by GISTIC, MITF activation score as obtained from our *MITF* gene signature, and the three categories classified according to the *BRAF* V600 mutation status and MITF activation score. Melanoma cells with a *BRAF* V600 mutated status and a high MITF activation score had higher sensitivity to PLX4720 than cells in the other two categories (Kruskal-Wallis test, p-value < 0.001).

We further examined the input data to explore the utility of *MITF* amplification and activation as a biomarker in addition to *BRAF* mutation status for predicting the response to a BRAF inhibitor. First, we detected relationships between the sensitivity profile to a BRAF inhibitor and genetic features, including *BRAF* mutation, *MITF* amplification, and expression profiles of MITF target genes, present in the JNMF input set (Fig. 4). Next, a MITF activation signature was generated based on the genes regulated by MITF in co-module #12, and the correlation between the MITF activation score and PLX4720 sensitivity profile in the JNMF input set was confirmed (rank correlation = 0.17, p-value = 0.0004, Fig. 4). This evidence suggests that JNMF can detect reasonable relationships hidden in multi-omics input data. Finally, we found that the activation status of MITF alone could serve as a useful novel biomarker to indicate the sensitivity to a BRAF inhibitor. Although a correlation between *BRAF* mutation and MITF activation was detected, they did not completely overlap in melanoma cells. However, tumours with both *BRAF* mutation and MITF activation were more sensitive to PLX4720 compared to tumours with *BRAF* mutation but without MITF activation (Fig. 5).

### Drug differentiation strategy using JNMF

Similar to biomarker discovery, an essential component of drug development is to understand the unique characteristics of individual drugs. Utilizing the parts-based representation of NMF can help to reveal important differences among drugs based on the results of JNMF.

Indeed, we detected remarkable differences in the JNMF results between two compounds with similar overall drug sensitivity profiles. For example, the RAS/BRAF/MEK signal transduction pathway is known to play an important role in tumour development in multiple cancer types. Thus, both BRAF and MEK inhibitors show similar biological effects on cancer cells. However, JNMF found that co-module #30, enriched with the features of colorectal tumours, was only sensitive to MEK inhibitor and did not exert efficacy to BRAF inhibitor (Fig. 3a). Both epidermal growth factor receptor (EGFR) and HER2 belong to the ERBB protein family. Thus, a single ERBB inhibitor has been developed for treating *EGFR*-mutated or *HER2*-amplified cancers. However, in the present analysis, co-module #5 showed that *HER2*-amplified and overexpressed breast cancers were sensitive to HER2 inhibition but were not sensitive to EGFR inhibition (Fig. 3b). Given that the present JNMF-based approach could detect critical, biologically verified differences in drugs, even between drugs with similar overall sensitivity profiles, it shows good promise for developing unique biomarker-based strategies to design novel compounds, with more reliability than currently available approaches.

## Discussion

The proposed JNMF method adapted to handle missing data allows for the integration of multiple genomic and pharmacological datasets, and our multi-dimensional clustering approach of JNMF could efficiently extract genetic alterations related to sensitivity profiles for specific compounds. Moreover, using simulated data, JNMF correctly detected co-modules or multi-dimensional clusters with predefined conditions containing 10% missing values.

By exploiting the parts-based representation of NMF, we successfully excluded the influence of other interacting factors to extract melanoma-specific features. Further, by combining known biological knowledge via pathway analysis, MITF downstream genes emerged as candidate biomarkers that can be interpreted based on a biological rationale rather than from simple prediction analysis derived from combining the influence of multiple features. Furthermore, use of a gene signature approach enabled estimation of the activation levels of the transcriptional regulator MITF and could predict responders and non-responders to BRAF inhibitors by integrating information of the known and candidate biomarkers. Specifically, tumours with both *BRAF* mutation and MITF activation were more sensitive to PLX4720 than tumours with *BRAF* mutation without MITF activation (Fig. 5). Therefore, activation of the BRAF/MITF axis might be a more appropriate biomarker for predicting the efficacy of a BRAF inhibitor than *BRAF* mutation alone, which is frequently used as a predictive biomarker in preclinical studies and clinical trials (Fig. S6).

Several studies have reported that MITF plays important roles in cell cycle progression by activating downstream genes in melanoma^21,22^. Consistent with our findings, forced expression of MITF conferred melanoma cells with sensitivity to BRAF/MEK inhibitors^23^. However, there is also a conflicting report that MITF activation might be involved in the mechanism of resistance to BRAF inhibition therapy^24^. Thus, our candidate biomarker should be evaluated in additional preclinical studies as well as in clinical trials.

With respect to lapatinib, JNMF clearly identified two major responder groups, breast cancer and lung cancer cells. This result is supported by the fact that lapatinib has been approved for use in clinical settings for treating HER2-positive breast cancer, and was shown to be effective in some types of lung cancer in a preclinical experiment^25^. Nevertheless, traditional chemotherapeutic agents did not show a peak in any of the co-modules since the majority of the included cell lines are sensitive to these agents. Therefore, it is still difficult to determine the characteristics of responder cell lines to broadly active compounds based on genomic and transcriptomic information.

Elastic net regression analysis was previously shown to effectively predict drug sensitivity based on several genetic features^3^. However, this approach selects one representative feature among highly correlated features, which makes it difficult to select the correlated MITF target genes simultaneously (Fig. 3). In the CCLE report, although the *BRAF* mutation emerged as a strong predictor of response to the PLX4720 compound, the MITF target genes and *MITF* amplification (except GAPDHS expression) were not selected as significant predictors, which is possibly because these features are sufficiently correlated with *BRAF* mutation.

As represented by the NCI DREAM challenge, drug sensitivity profiles are typically predicted using comprehensive multi-omics data with incorporation of available biological knowledge. However, there is still a substantial gap between such basic research and companion diagnostic development through clinical trials. One possible reason for this limitation is that a predictive model must be sufficiently interpreted and explained to both the patients and doctors when used for patient selection in a clinical setting. Thus, a future challenge might be how to best explain a diagnostic result derived from multi-omics biomarkers in a clinical situation. Accordingly, in addition to refining multi-omics itself, another crucial task for practical realization is to establish a user-friendly interpretable format for outputting the data.

Prediction of missing values by JNMF is expected to be a useful feature for inferring a mutation status or drug sensitivity profile from other types of multi-omics information such as expression profiles. Given that our JNMF approach could efficiently predict missing values from simulated data, and the clustering was useful for detecting co-modules for datasets containing missing values, it is necessary to further verify whether this missing value prediction using JNMF is applicable to actual data.

There are several successful examples of drug development using a biomarker-based strategy, including the development of third-generation EGFR inhibitors and PARP inhibitors. Molecular and clinical profiles of the investigational drugs themselves were similar with respect to the mode-of-action (MOA). However, the patient stratification strategy for clinical trials based on the biomarker differed for different drugs, signifying the importance of a useful predictive biomarker for successful drug development. The proposed approach of incorporating the JNMF method with biomarker selection could help to increase the success rate of drug development by providing a differentiated and superior biomarker strategy compared with those used previously for investigational drugs with a similar MOA.

Overall, we have presented an NMF-based unsupervised approach for discovering useful biomarkers using *in vitro* multi-dimensional cell line data. The generated JNMF co-modules are based on meta-profiles derived from whole genomic and pharmacological data. Therefore, candidate biomarkers are derived from the relationship between multi-therapeutic targets and multi-genetic alterations. This concept is quite different from a single therapeutic model of a compound such as ANOVA and elastic net regression. Our JNMF simultaneously detects differences between compound profiles and should thus be useful for exploring drug development strategies while revealing genomic characteristics or therapeutic targets for unmet medical needs. Furthermore, the proposed biomarker discovery scheme will be useful for finding pharmacodynamic biomarkers and for MOA analysis at the preclinical stage. Together, this approach and similar developments should accelerate translational research and mining for clinical significance using clinical response data.

## Methods

### Data summarization, normalization, and preprocessing

Mutation, copy number alterations, mRNA expression, and compound sensitivity profiles were collected from the CCLE (Table S1). Mutation profiles were converted into binary data of mutant type (1) or wild type (0) for each gene. Copy number profiles generated by the GISTIC algorithm^26^ were divided into amplification (GISTIC score: +2) and deletion (GISTIC score: −2) profiles to obtain respective binary datasets of amplification and deletion. mRNA expression profiles were normalized from the log2 intensity values to a 0–1 score for each gene. The concentration at which the drug response reached an absolute inhibition of 50% (IC_50_) was used as an index of the compound sensitivity. The IC_50_ values were converted to a score of insensitive (0) and sensitive (1) according to the following formula:

1 $${\rm{Normalized}}\,{{\rm{IC}}}_{50}=\frac{{x}_{max}-x}{{x}_{max}-{x}_{min}},$$

where *x* indicates the IC_50_ value and *x*_*max*_ and *x*_*min*_ are the maximum and minimum IC_50_ values of a compound among the CCLE cell lines tested.

A tumour-type binary matrix was also obtained based on the CCLE lineage information. Thus, a total of six 0–1-normalized matrices *X*_1_, *X*_2_, …, *X*_6_ were obtained: compound sensitivity, mutation, amplification, copy number deletion, mRNA expression, and tumour type. A total of 504 cell lines with sensitivity data for at least one compound were subjected to subsequent analysis.

### JNMF and mask matrix

JNMF was performed for ***N*** matrices. The objective function of JNMF is a squared Euclidean error function and formulated as

2 $${\rm{\min }}\,\sum _{I=1}^{N}{\Vert {X}_{I}-W{H}_{I}\Vert }_{F}^{2}$$

where *X*_*I*_ is an *m* × *n*_*I*_ input matrix, *W*, *H*_*I*_ represents *m* × *k*, *k* × *n*_*I*_ output matrices, *k* is the factorization rank of JNMF, and *F* shows Frobenius norm. *N* = 3 was used for simulated data and *N* = 6 matrices from CCLE data were obtained. Then, the standard multiplicative update rules were selected for NMF calculation as follows:

3 $${W}_{ia}\leftarrow {W}_{ia}\frac{{({\sum }_{J=1}^{N}({X}_{J}{H}_{J}^{T}))}_{ia}}{{(W{\sum }_{K=1}^{N}({H}_{K}{H}_{K}^{T}))}_{ia}}$$

4 $${({H}_{I})}_{a\mu }\leftarrow {({H}_{I})}_{a\mu }\frac{{({W}^{T}{X}_{I})}_{a\mu }}{{({W}^{T}W{H}_{I})}_{a\mu }},\,I=1,\cdots ,N.$$

The NMF procedure was modified to handle matrices containing missing values using a mask matrix. In brief, we used a weighted NMF approach^27^. The mask matrix *M* has the same dimensions as the input matrix *X*, with 1 in the case where there is a value in *X* in each matrix element and 0 in the case where there is not. For the execution of JNMF, the product of each matrix element of *X* and *M* is obtained so that the normal JNMF calculation is executed when the value of *X* exists and is ignored when the value of *X* does not exist.

5 $${\rm{\min }}\,\sum _{I=1}^{N}{\Vert {M}_{I}\,\circ \,({X}_{I}-W{H}_{I})\Vert }_{F}^{2}$$

6 $${W}_{ia}\leftarrow {W}_{ia}\frac{{({\sum }_{J=1}^{N}(({M}_{J}\circ {X}_{J}){H}_{J}^{T}))}_{ia}}{{({\sum }_{K=1}^{N}({M}_{K}\circ (W{H}_{K}){H}_{K}^{T}))}_{ia}}$$

7 $${({H}_{I})}_{a\mu }\leftarrow {({H}_{I})}_{a\mu }\frac{{({W}^{T}({M}_{I}\circ {X}_{I}))}_{a\mu }}{{({W}^{T}({M}_{I}\circ (W{H}_{I})))}_{a\mu }},\,I=1,\cdots ,\,N,$$

where $A\,\circ \,B=[{a}_{ij}{b}_{ij}]$ represents the Hadamard product.

This JNMF update procedure for *W* and each *H*_*I*_ is executed at 5,000 iterations with an appropriate factorization rank *k*, and a convergence is observed (Fig. S2). It is repeated for a prescribed number of times $T$ and a result is selected in which the difference between *X* and *WH* is minimal among the *T* results. In addition, a consensus matrix and its cophenetic correlation coefficient from all *T* results were obtained to examine the reproducibility.

### Pathway and gene signature analyses

IPA^16^ was used to predict the activated pathways in JNMF co-modules. IPA upstream and IPA protein-protein interaction analyses are used to elucidate the common regulators of gene sets in the JNMF co-module. For a gene set (***n***_***g***_ genes), the average expression level was taken as the gene signature representing pathway activation according to the following formula:

8 $${\rm{Signature}}\,{{\rm{Score}}}_{i}=\,\frac{1}{{n}_{g}}\sum _{j}^{{n}_{g}}(\frac{{x}_{ij}-\mathop{{\rm{\min }}}\limits_{i}{x}_{ij}}{\mathop{{\rm{\max }}}\limits_{i}{x}_{ij}-\mathop{{\rm{\min }}}\limits_{i}{x}_{ij}})$$

where *x*_*ij*_ indicates the mRNA expression level of a gene *j* in a cell *i*.

Signature score is calculated for ***n***_***g***_ genes in mRNA profiles *X*_5_, obtained by integrating the JNMF co-module and IPA knowledge.

### Data availability

The data that support the findings of this study are available on the Cancer Cell Line Encyclopedia (https://portals.broadinstitute.org/ccle)^3^ and the cBioPortal for Cancer Genomics (http://www.cbioportal.org/)^28,29^.

## Electronic supplementary material

Supplementary Figures 1-6

Supplementary Tables 1,2

## References

[1] Collins FS, Varmus H (2015). A new initiative on precision medicine. The New England journal of medicine.

[2] Reck M (2016). Pembrolizumab versus Chemotherapy for PD-L1-Positive Non-Small-Cell Lung Cancer. The New England journal of medicine.

[3] Barretina J (2012). The Cancer Cell Line Encyclopedia enables predictive modelling of anticancer drug sensitivity. Nature.

[4] Garnett MJ (2012). Systematic identification of genomic markers of drug sensitivity in cancer cells. Nature.

[5] Haibe-Kains B (2013). Inconsistency in large pharmacogenomic studies. Nature.

[6] Pharmacogenomic agreement between two cancer cell line data sets. *Nature* 528, 84–87, 10.1038/nature15736 (2015). 10.1038/nature15736 PMC6343827 26570998

[7] Costello JC (2014). A community effort to assess and improve drug sensitivity prediction algorithms. Nature biotechnology.

[8] Schmidt KT, Chau CH, Price DK, Figg WD (2016). Precision Oncology Medicine: The Clinical Relevance of Patient-Specific Biomarkers Used to Optimize Cancer Treatment. Journal of clinical pharmacology.

[9] Lee DD, Seung HS (1999). Learning the parts of objects by non-negative matrix factorization. Nature.

[10] Nik-Zainal S (2012). Mutational processes molding the genomes of 21 breast cancers. Cell.

[11] Alexandrov LB, Nik-Zainal S, Wedge DC, Campbell PJ, Stratton MR (2013). Deciphering signatures of mutational processes operative in human cancer. Cell reports.

[12] Tamayo P (2007). Metagene projection for cross-platform, cross-species characterization of global transcriptional states. Proceedings of the National Academy of Sciences of the United States of America.

[13] Zhang S (2012). Discovery of multi-dimensional modules by integrative analysis of cancer genomic data. Nucleic acids research.

[14] Yang Z, Michailidis G (2016). A non-negative matrix factorization method for detecting modules in heterogeneous omics multi-modal data. Bioinformatics.

[15] Khatri P, Sirota M, Butte AJ (2012). Ten years of pathway analysis: current approaches and outstanding challenges. PLoS computational biology.

[16] Kramer A, Green J, Pollard J, Tugendreich S (2014). Causal analysis approaches in Ingenuity Pathway Analysis. Bioinformatics.

[17] Singh A (2009). A gene expression signature associated with “K-Ras addiction” reveals regulators of EMT and tumor cell survival. Cancer cell.

[18] Itadani H, Mizuarai S, Kotani H (2008). Can systems biology understand pathway activation? Gene expression signatures as surrogate markers for understanding the complexity of pathway activation. Current genomics.

[19] Yang H (2010). RG7204 (PLX4032), a selective BRAFV600E inhibitor, displays potent antitumor activity in preclinical melanoma models. Cancer research.

[20] Bollag G (2012). Vemurafenib: the first drug approved for BRAF-mutant cancer. Nature reviews. Drug discovery.

[21] Garraway LA (2005). Integrative genomic analyses identify MITF as a lineage survival oncogene amplified in malignant melanoma. Nature.

[22] Levy C, Khaled M, Fisher DE (2006). MITF: master regulator of melanocyte development and melanoma oncogene. Trends in molecular medicine.

[23] Ji Z (2015). MITF Modulates Therapeutic Resistance through EGFR Signaling. The Journal of investigative dermatology.

[24] Johannessen CM (2013). A melanocyte lineage program confers resistance to MAP kinase pathway inhibition. Nature.

[25] Moy B, Kirkpatrick P, Kar S, Goss P (2007). Lapatinib. Nature reviews. Drug discovery.

[26] Mermel CH (2011). GISTIC2.0 facilitates sensitive and confident localization of the targets of focal somatic copy-number alteration in human cancers. Genome biology.

[27] Li Y, Ngom A (2013). The non-negative matrix factorization toolbox for biological data mining. Source code for biology and medicine.

[28] Gao J (2013). Integrative analysis of complex cancer genomics and clinical profiles using the cBioPortal. Science signaling.

[29] Cerami E (2012). The cBio cancer genomics portal: an open platform for exploring multidimensional cancer genomics data. Cancer discovery.

