# Supplementary material for: Biomarker discovery by integrated joint non-negative matrix factorization and pathway signature analyses
Source: Sci Rep. 2018 Jun 27;8:9743. doi: 10.1038/s41598-018-28066-w (PMC6021419; doi:10.1038/s41598-018-28066-w)
Supplement: Supplementary file 1 — Supplementary Figures 1-6 [file 41598_2018_28066_MOESM1_ESM.pdf]

# Biomarker discovery by integrated joint non-negative matrix factorization and pathway signature analyses

Naoya Fujita[1, 2], Shinji Mizuarai[2], Katsuhiko Murakami[1], and Kenta Nakai[1,*]

[1]Human Genome Center, the Institute of Medical Science, the University of Tokyo, Tokyo, Japan

[2]Discovery and Preclinical Research Division, Taiho Pharmaceutical Co., Ltd., Tsukuba, Japan

*Corresponding author

Phone: +81-3-5449-5131

E-mail: knakai@ims.u-tokyo.ac.jp

**Supplementary Figures 1-6**

**Fig. S1**

Fig. S1a

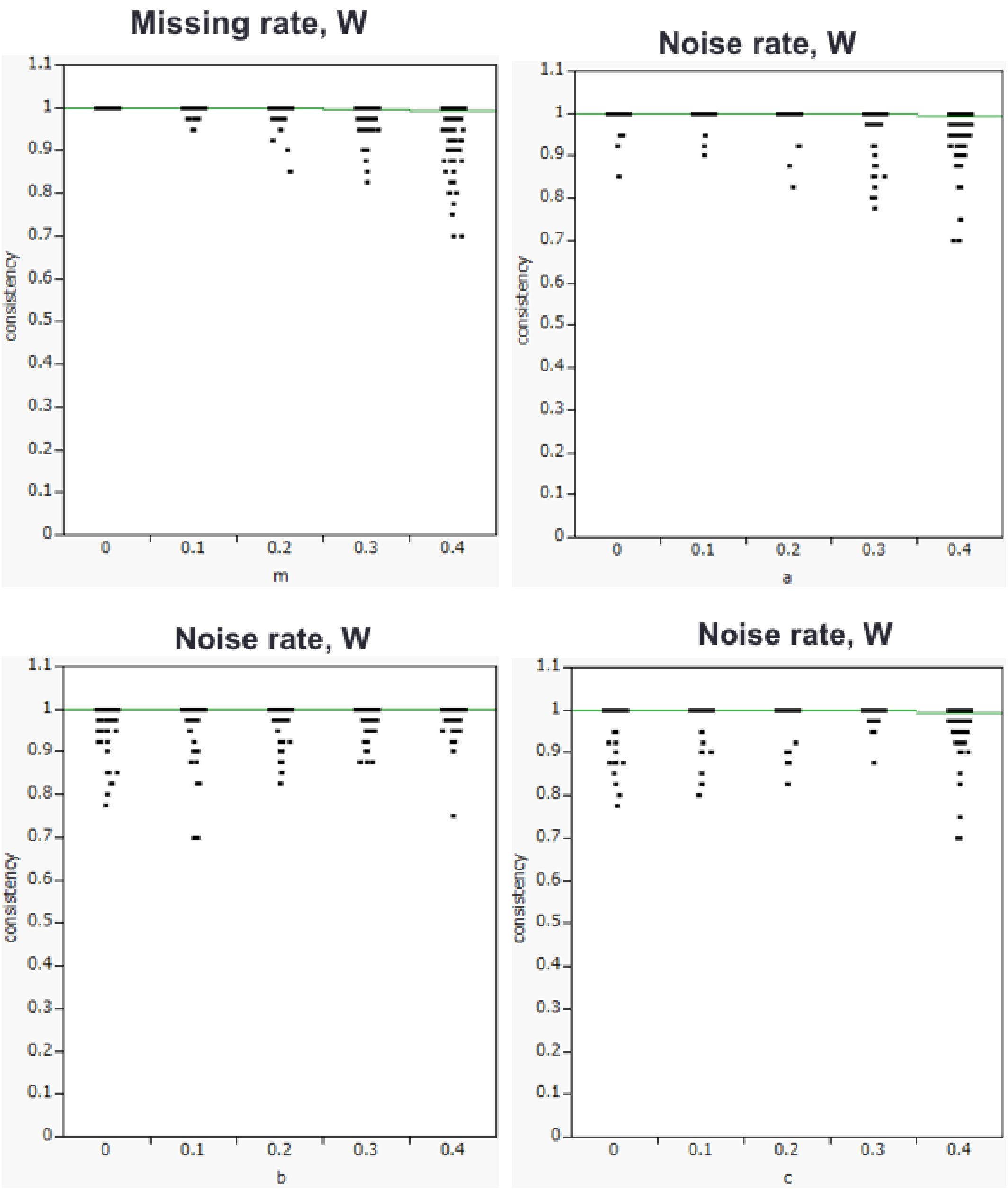

Fig. S1b

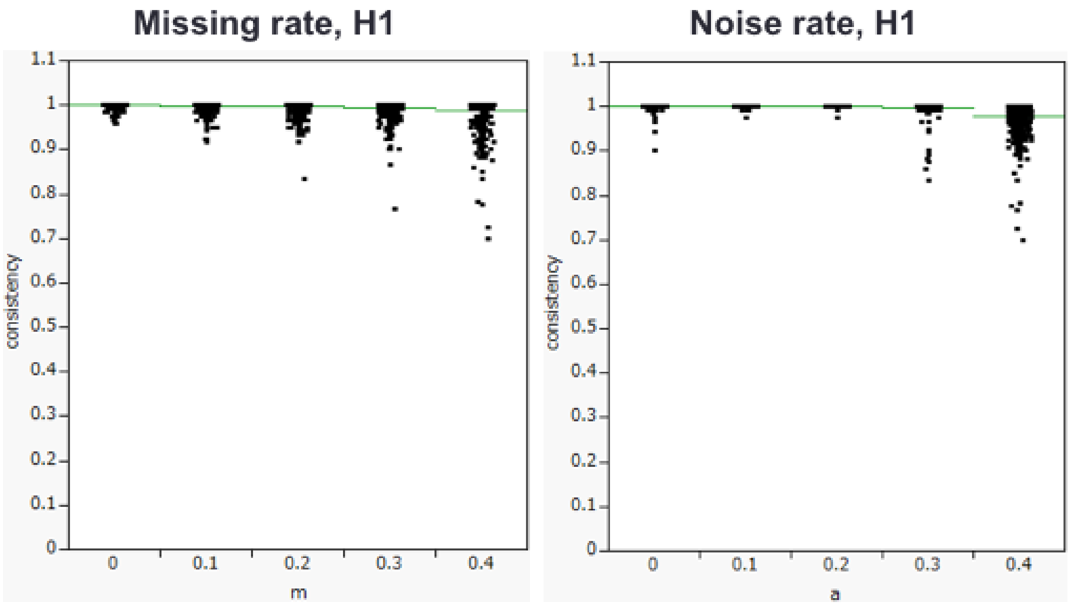


Fig. S1c

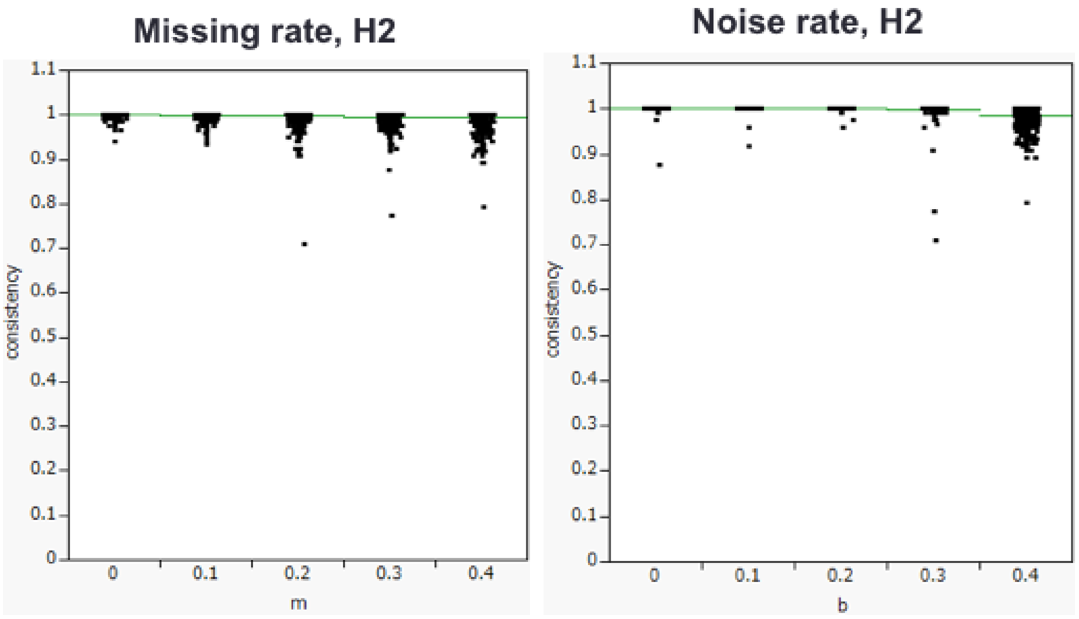

Fig. S1d

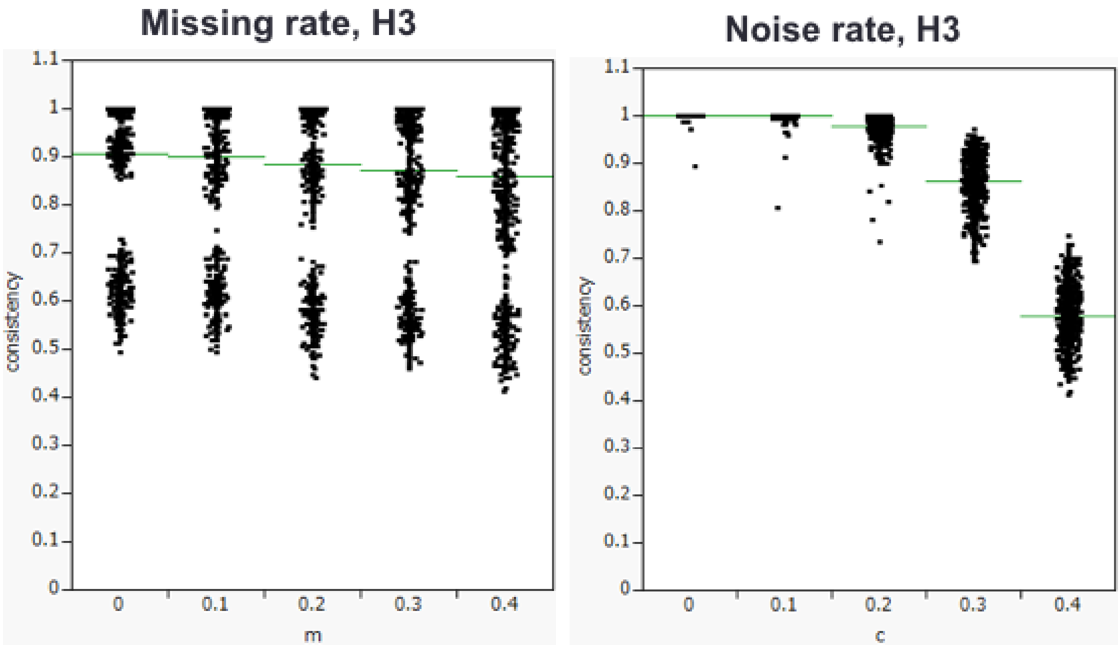


**Fig. S1| Impact of noise and missing values on JNMF output matrices**

**(a)** Relationship between missing and noise parameters and consistency of predefined clusters on $W$. The regression model is $y_W = 1.01 - 0.01a$-$0.00b$-$0.01c$-$0.01m$ **(b)** Relationship between missing and noise parameters $m, a$ and consistency of predefined clusters on $H_1$. The regression model is $y_{H1} = 1.01 - 0.04a + 0.00b + 0.00c$-$0.02m$ **(c)** Relationship between missing and noise parameters $m, b$ and consistency of predefined clusters on $H_2$. The regression model is $y_{H2} = 1.01 - 0.00a + 0.03b$-$0.01c$-$0.02m$ **(d)** Relationship between missing and noise parameters $m, c$ and consistency of predefined clusters on $H_3$. The regression model is $y_{H3} = 1.11 - 0.03a$-$0.02b$-$0.98c$-$0.12m$

**Fig. S2**

Fig. S2a

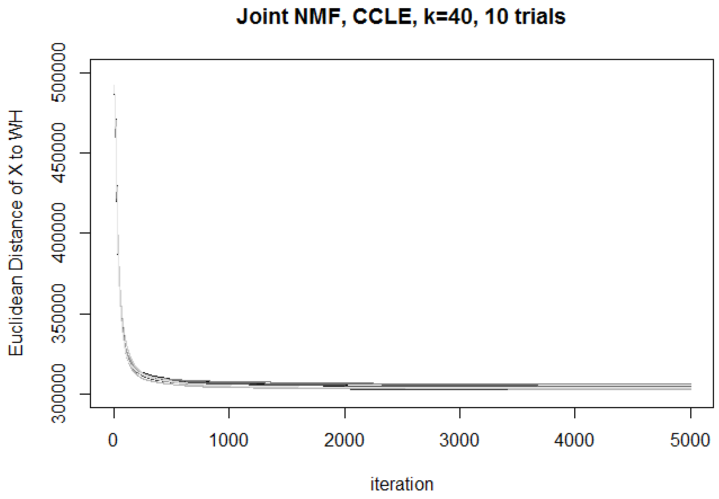


Fig. S2b

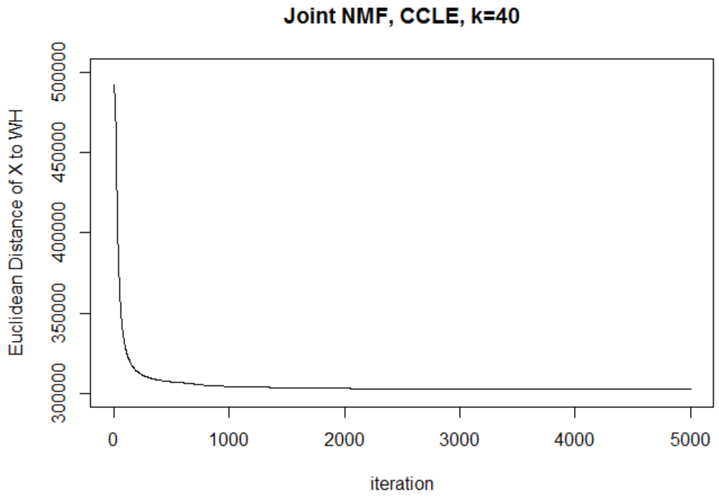


**Fig. S2| Convergence of JNMF objective function**

**(a)** Each gray and black line shows trajectory of the JNMF residuals, computed with the objective function. **(b)** The trajectory of best result reached to the smallest objective function value among the 10 trials.

**Fig. S3**

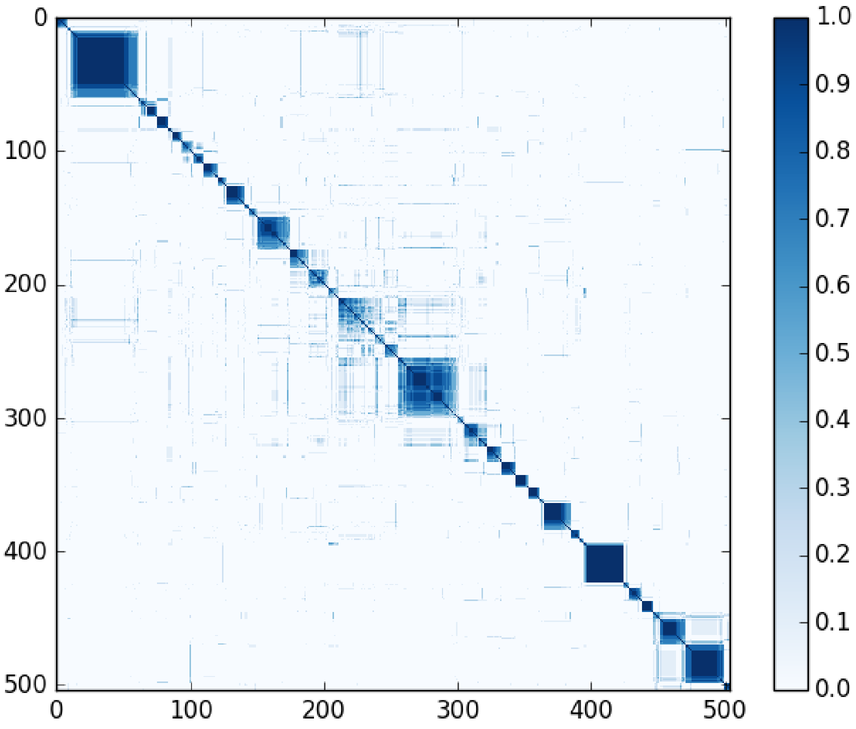


**Fig. S3| Consensus matrix of the cancer cell line encyclopedia data**

This consensus matrix shows reproducibility of JNMF output $W$ in ten replicate trials. Both axes show cell lines in a cluster order. Blue components shows perfect grouping as in a cluster and white shows these cells are assigned in different clusters. Cophenetic correlation coefficient = 0.91.

**Fig. S4**

IPA_MITF_melanoma_module12

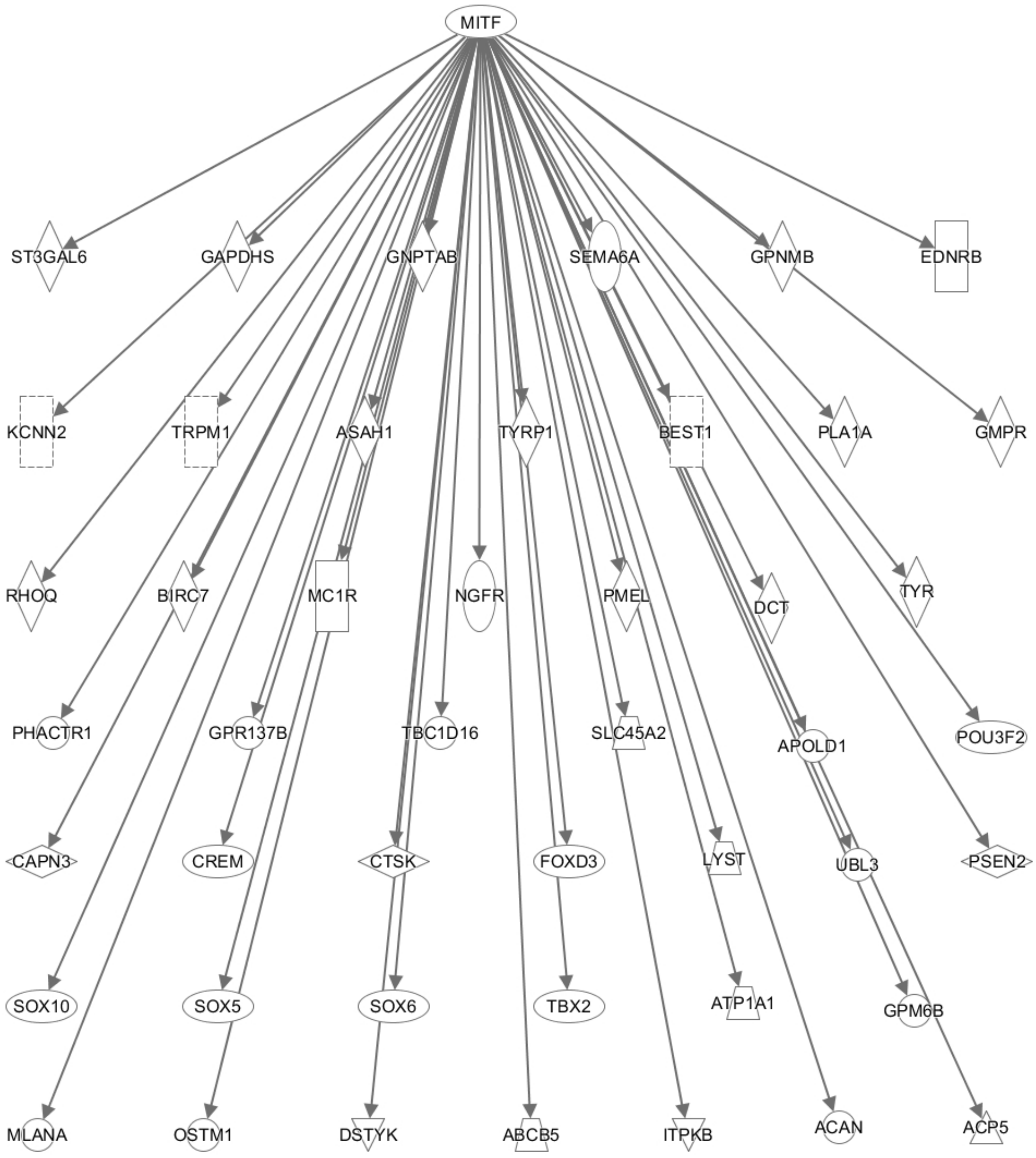


© 2000-2017 QIAGEN. All rights reserved.

**Fig. S4| MITF target genes in a co-module (#12)**

Upstream analysis in the Ingenuity Pathway Analysis tool detected 47 genes out of 354 genes in a module (#12) as MITF downstream genes. Solid arrows mean that expression levels of these 47 mRNAs are reported as directly regulated by the transcription factor MITF.

**Fig. S5**

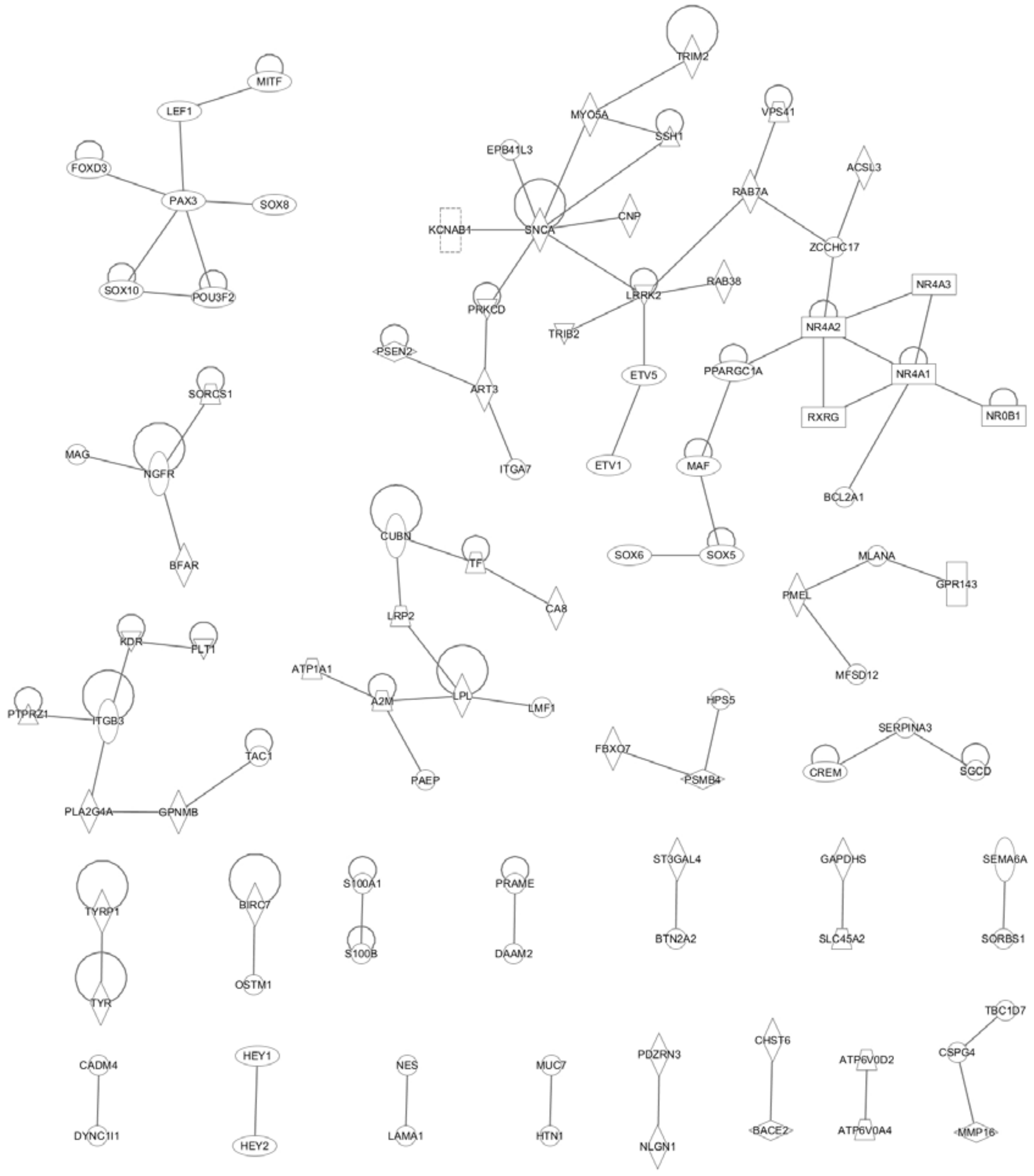


© 2000-2018 QIAGEN. All rights reserved.

**Fig. S5| Protein-protein interaction networks in a co-module (#12)**

Protein-protein interaction analysis in the Ingenuity Pathway Analysis tool detected a sub-network of melanoma-related transcription factors, MITF, PAX3, SOX8, SOX10, POU3F2, FOXD3, and LEF1, out of 354 genes in a module (#12). Edges are based on the evidence of protein-protein interactions between nodes from literature.

**Fig. S6**

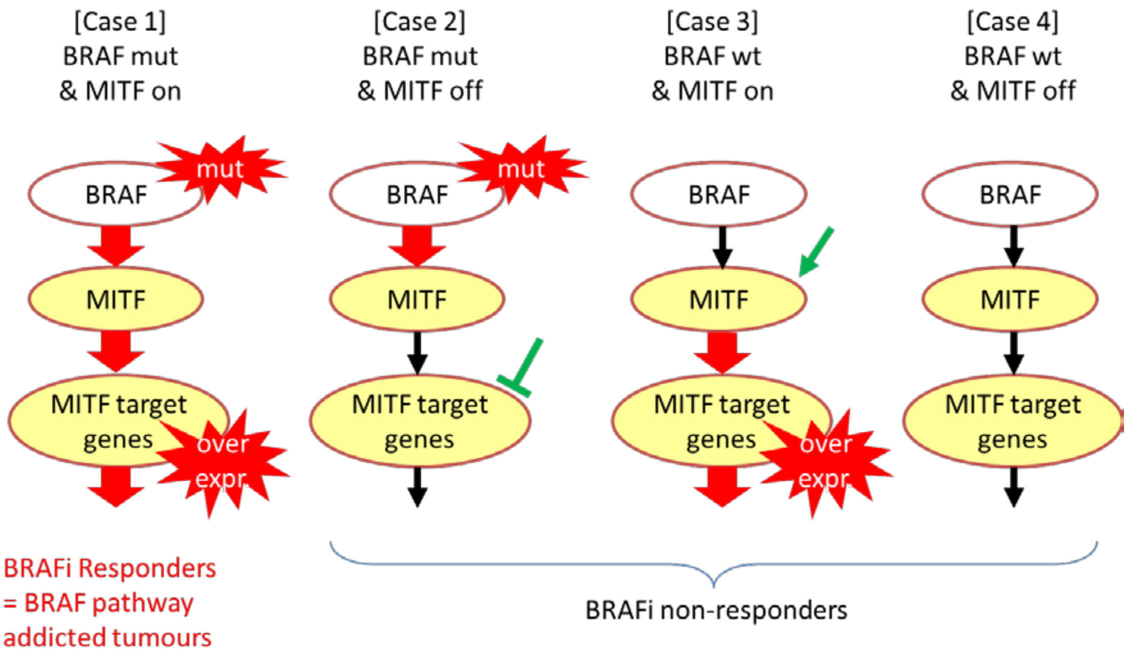


**Fig. S6| MITF activation status as the additional biomarker for BRAFi**

The illustration describes activation of the BRAF/MITF axis might be a more appropriate biomarker for predicting the efficacy of a BRAF inhibitor than BRAF mutation alone.
